# Case Series: Recovery of Chemotherapy-Related Acute Heart Failure by the Combined Use of Sacubitril Valsartan and Wearable Cardioverter Defibrillator: A Novel Winning Combination in Cardio-Oncology

**DOI:** 10.3389/fcvm.2022.801143

**Published:** 2022-03-01

**Authors:** Maria Laura Canale, Katia Coviello, Gianluca Solarino, Jacopo Del Meglio, Federico Simonetti, Elio Venturini, Andrea Camerini, Nicola Maurea, Irma Bisceglia, Carlo Tessa, Giancarlo Casolo

**Affiliations:** ^1^Division of Cardiology, Azienda USL Toscana Nord-Ovest, Versilia Hospital, Lido di Camaiore, Italy; ^2^Hematology, Azienda USL Toscana Nord-Ovest, Versilia Hospital, Lido di Camaiore, Italy; ^3^Cardiac Rehabilitation Unit, Azienda USL Toscana Nord-Ovest, Civil Hospital, Cecina, Italy; ^4^Medical Oncology, Azienda USL Toscana Nord-Ovest, Versilia Hospital, Lido di Camaiore, Italy; ^5^S.C. Cardiologia, Istituto Nazionale Tumori, IRCCS Fondazione G. Pascale, Naples, Italy; ^6^Servizi Cardiologici Integrati, Azienda Ospedaliera San Camillo-Forlanini, Rome, Italy; ^7^Division of Radiology, Azienda USL Toscana Nord-Ovest, Nuovo Ospedale Apuano, Massa, Italy

**Keywords:** sacubitril/valsartan, wearable cardioverter defibrillator, cardio-oncology, heart failure, anthracyclines

## Abstract

Effective anticancer treatments have dramatically improved the outcome of patients with cancer, but cardiac toxicity reduces their clinical efficacy in a non-negligible percentage of patients. Sacubitril/valsartan is a new paradigm in the treatment of chronic heart failure, with a reduced ejection fraction due to the enhancement of natriuretic peptides' properties when coupled with a blocking effect on the angiotensin II type 1 (AT1) receptors. As with other clinical conditions of heart failure with potentially reversible declines in cardiac function, a wearable cardioverter defibrillator (WCD) is a valid tool for protection against sudden death until recovery occurs. We report a case series of four patients with chemotherapy-related acute cardiac failure with severely reduced cardiac function. They were successfully treated with sacubitril/valsartan while being protected from malignant arrhythmias using a wearable cardioverter defibrillator until the recovery of cardiac function. Sacubitril/valsartan was confirmed to be effective in anthracycline-related cardiac toxicity and the wearable cardioverter defibrillator should be considered as a support tool even in the oncology patient.

## Introduction

Improvements in anti-cancer global strategy have resulted in better outcomes for a large part of patients with cancer, with many of them experiencing remission or long-term survival. Cardiovascular disease is the second leading cause of death in cancer survivors, preceded only by the cancer-related mortality itself (1). Systolic dysfunction is a well-documented and dose-dependent side effect of anthracyclines, which, in many cases, can be reversed with the introduction of heart failure therapy (2). Angiotensin-converting enzyme (ACE)-inhibitors, or angiotensin receptor blockers, together with beta-blockers, are the cornerstone of treatment of cancer therapy-related cardiac dysfunction (3). Sacubitril/valsartan (S/V) is a combined neprilysin inhibitor and angiotensin AT1 receptor blocker, which is approved for the treatment of chronic heart failure, with a reduced ejection fraction (4). No data from the pivotal PARADIGM-HF trial on cancer therapy-related cardiac dysfunction is available as these patients, although not formally excluded, have not been enrolled. Recently, some retrospective real-world data on S/V use in patients with reduced ejection fraction after chemotherapy showed an improvement in myocardial performance and reverse remodeling, but sound evidence is still lacking (5–8). Patients with heart failure (HF) and reduced ejection fraction (HFrEF) have a major risk of malignant arrhythmias and sudden cardiac death. The use of a wearable cardioverter defibrillator (WCD) is a valid tool for protection against sudden death in cases of potentially reversible cardiac damage. In patients with cancer, a WCD can be useful for those with potentially reversible cardiac toxicity during the implementation of evidence-based medical therapy. Interestingly, planned radiotherapy does not represent a contraindication to WCD since it can be easily removed to avoid interference between ionizing radiation and device function (9).

Cardiac magnetic resonance (CMR) represents the gold standard in cardiac function assessment and can be used for an early-stage screening of heart damage. Its role in tissue characterization can help eliminate other possible causes of myocardial dysfunction when cardiac toxicity is suspected. For this purpose, T1 and T2 mapping, in addition to late gadolinium enhancement (LGE), are very useful tools (10). We collected a case series of four patients with acute heart failure and deeply depressed EF secondary to anthracyclines or carfilzomib cardiotoxicity. We investigated the use of S/V to rescue the cardiac systolic function, together with the use of WCD, while waiting for the effects of medical therapy.

## Cases Presentation

### Case 1

A 57-yo man with a malignant abdominal desmoid tumor, with long-term anthracyclines treatment (cumulative dose of 600 mg/m^2^), and with a regular clinical and echocardiographic follow-up. The patient was hospitalized in November 2019 for acute heart failure with reduced ejection fraction (EF 25%) 1 month after the end of chemotherapy. The event occurred 8 months after the treatment started and 5 months after crossing the threshold dose of 300 mg/m^2^. The patient started the anthracyclines therapy after a long period of hospitalization due to complications from abdominal surgery and a significant weight loss. No coronary lesions were detectable on coronary angiography. The CMR findings were an increased biventricular size and a severe biventricular dysfunction, with left systolic ejection fraction (LVEF) of 25% and right ventricle (RV) EF of 40%, increased native T1 signal (1,095 ms vs. normal value of 950 ± 21 ms) with a marked increase of extracellular volume (ECV) (40% vs. normal value of 26 ± 4%), but no LGE was found. Laboratory findings showed normal high-sensitive C-reactive protein (hs-CRP) levels and glomerular filtration rate. High-sensitive troponin T (hs-TnT) was elevated at admission time (190 pg/ml), with a slight decline during the in-hospital stay (162 pg/ml). The N-terminal prohormone of natriuretic peptide (NT-proBNP) levels was clearly elevated at baseline (9,752 pg/ml), but we observed a reduction during hospitalization to 7,200 pg/ml at discharge. During the follow-up, the NT-proBNP levels continued to decrease to 530 pg/ml within the year. Given the severe LV dysfunction, the patient was discharged with heart failure therapy (ACE-inhibitor, beta-blocker, anti-aldosterone therapy) and a WCD. At the first follow-up visit 10 days later, ACE-inhibitor was replaced with S/V due to persistent systolic dysfunction. After 3 months, the LVEF increased from 25 to 40% with the reverse remodeling of ventricular size and WCD was discontinued. The patient compliance to WCD was high with a median of 22.4 h/day. No significant arrhythmias were detected. Echocardiography findings were confirmed at 6 months by CMR (LVEF 43% and RVEF 51%), and while the native myocardial T1 signal decreased, it was not yet normal (1,051 vs. 1.095 ms). At the one-year follow-up visit, the LVEF had improved to 45% (Table 1).

**Table 1:** Summary of clinical and imaging characteristics of the presented cases.

	**Case 1**	**Case 2**	**Case 3**	**Case 4**
Age	57	65	83	65
Cancer site	Desmoid	Breast	Multiple Myeloma	Breast
Drug	Anthra	Anthra Trastuzumab	Carfilzomib	Anthra
total dose (mg/m^2^)	600	360 na	na	540
Admission LVEF	25%	30%	25%	25%
LVEF after S/V	45%	48%	48%	46%
Recovery time	3 months	3 months	2 weeks	3 months
Baseline T1 msec	1,095	1,040	1,066	986
T1 after S/V msec	1,051	1,000	na	na
Baseline ECV	40%	na	32%	23%
ECV after S/V	na	28%	na	na
LGE (yes/no)	No	No	Yes (non ischemic)	No

*Anthra, anthracyclines; LVEF, left ventricular ejection fraction; S/V, sacubitril/valsartan; ECV, extracellular volume; LGE, late gadolinium enhancement; na, not applicable/not available*.

### Case 2

A 65-year-old woman, with early left breast cancer, was treated with surgery and radiotherapy in 2006, and adjuvant epirubicin (cumulative dose 360 mg/m^2^) plus cyclophosphamide followed by trastuzumab for 1 year, ending in 2007. In September 2020, the patient was hospitalized for acute heart failure, with EF at 30%, and a coronary computed tomography (CT) that documented the absence of coronary artery disease. The patient reported smoking habits and hypertension in her medical history. The Hs-CRP and hs-TnT values and the glomerular filtration rate were all within the normal range, while NT-proBNP levels were elevated at admission (2,636 pg/ml), but during hospitalization, they rapidly decreased to 863 pg/ml at discharge and normalized at 3 months follow-up. The CMR showed an LV dilatation with severe impairment of contractile function (LVEF 32%) and a mild RV failure (RVEF 48%); native myocardial T1 signal was increased (1,040 ms) while LGE was absent. The patient was discharged with WCD and heart failure therapies including beta-blocker, anti-aldosterone therapy, and S/V that was later replaced by ramipril (2.5 mg/day) due to hypotension. After 3 months, the CMR highlighted a normalized left ventricular size and right ventricular function (RVEF 69%), left ventricle systolic function improvement (LVEF 48%), and a native myocardial T1 signal decreased to 1,000 ms with respect to baseline 1,040 ms with normal ECV (28%). No LGE was detectable, but septal edema was still present in the T2 mapping. The WCD was discontinued due to LVEF recovery (Table 1). Patient compliance to WCD was high with a median of 23.4 h/day with no significant arrhythmias detected.

### Case 3

An 83-year-old man with advanced multiple myeloma was treated with a first-line bortezomib-melfalan-prednisone combination in 2018, and with carfilzomib in December 2020 after the disease relapse. After 1 week since carfilzomib was started, the patient was hospitalized for acute heart failure with severe biventricular dysfunction (LVEF 25%). The patient presented with a reduced glomerular filtration rate and elevated hs-CRP levels (42 mg/L). The filtration rate remained unchanged during the in-hospital stay, while hs-CRP decreased to 18 mg/L. A high-sensitive troponin T was elevated at admission time (130 pg/ml) with a trend in reduction (90 pg/ml). The baseline NT-proBNP levels were clearly elevated (13,735 pg/ml), with a sharp reduction during hospitalization (1,137 pg/ml at discharge). A coronary CT (coronary angiography was excluded due to renal impairment) showed diffuse and severe coronary atherosclerosis without obstructive coronary disease. The patient was started on heart failure therapy with beta-blocker and S/V; after 2 weeks from therapy, introductory CMR was performed and has shown the normal biventricular dimensions and right ventricular function (RVEF 60%), left ventricular systolic function improvement (LVEF 45 vs. 25%), interventricular septal and lateral wall abnormal native T1 signal (1,000 and 1,066 ms, respectively), and an increased ECV (32%) and a mid-wall septal LGE with a non-ischemic pattern. At the one-month follow-up, LVEF had further improved to 48% (Table 1).

### Case 4

A 65-year-old woman with early left breast cancer was treated with adjuvant anthacycline-based chemotherapy (epirubicin with a cumulative dose of 540 mg/m^2^), followed by radiotherapy (dose 50Gy) in 2003. In January 2021, the patient reported dyspnea for mild effort. This patient also reported a history of smoking and hypertension. Chest X-Ray showed a pleural effusion, and the echocardiography documented a dilated left ventricle with severe systolic dysfunction (EF 25%) leading to hospitalization. The admission hs-CRP levels and hs-TnT were slightly elevated but remained in a stable trend during hospitalization. The filtration rate showed a baseline reduction that stayed unchanged during observation. As expected, the NT-proBNP levels were clearly elevated at baseline (15,600 pg/ml) but we observed a sharp and fast reduction (2,300 pg/ml at discharge). No coronary artery disease was found at coronary angiography. CMR confirmed an increased left ventricular size, severe biventricular systolic dysfunction (LVEF 27% and RVEF 29%), and no significant LGE. Heart failure therapy, with beta-blocker and anti-aldosterone therapy plus S/V, was started together with SGLT2 inhibitor empagliflozin for the previously undiagnosed diabetes. The patient was discharged with WCD. At the first follow-up visit 1 month later, LV size was normal, and the ejection fraction improved to 35%. At the three-month follow-up visits, LVEF had improved to 46%, and WCD was discontinued (Table 1). Once more, compliance to WCD was high with a median of 23.5 h/day. No significant arrhythmias were detected.

## Discussion

In this case series, we investigate the role of early S/V use in chemotherapy-induced cardiomyopathy, coupled with the use of WCD as a bridging therapy while waiting for ventricular function recovery. The CMR played a major role in confirming the echocardiographic data, in ruling out the ischemic etiology, (11) and in better defining the cardiac damage (10).

Chemotherapy-related heart failure may present a wide time range from the start of treatment, and this fact is clearly shown in our case series. In cases 2 and 4, the HF symptoms showed up to 13 and 18 years from anticancer therapy, respectively, while the time interval was shorter for case 1; while in case 3, symptoms appeared just 1 week after the treatment started. In cases with a long time interval, treatment-related cardiac damage acts as an additional cardiovascular risk factor (12). Acute and/or early forms are less characterized, although also include an immune-inflammatory pattern with a widespread cell death-mediated myocardial damage. This hypothesis perfectly fits with case 3. The first case was probably presented with immunosuppression that is related to malnutrition and prolonged hospitalization, which makes the hypothesis of immune-inflammatory myocardial damage less likely. Irrespective of the early or late clinical presentation, while our cases showed a prompt recovery of cardiac function, they should undergo a tailored cardiological follow-up schedule and a multidisciplinary approach if a new oncological treatment is needed.

Chemotherapy-related cardiomyopathy, with functional impairment, may be successfully treated with an HF therapy, particularly when inhibitors of the renin-angiotensin system are used. Although these patients were not enrolled in the PARADIGM-HF trial (4) due to a history of chemotherapy-related HF over the last 12 months, the use of S/V in the setting of cardiac dysfunction secondary to chemotherapy is an intuitive therapeutic opportunity (13). However, all currently available data comes from case reports/series and retrospective analyses, and prospective validation of its use is still lacking. A retrospective multicenter registry showed that S/V was well-tolerated and could improve the myocardial function and the structure in patients with cancer and with chemotherapy cardiomyopathy (6). Positive effects of S/V on cardiac structure and function in chemotherapy-damaged hearts were also reported recently. A group of patients underwent CMR at baseline, and after 3 months from the beginning of S/V therapy, the findings were consistent with the reverse remodeling of LV volumes, improvement of LVEF, and reduction of NTpro-BNP levels (14).

A meta-analysis highlighted the effect of S/V on reverse cardiac remodeling in patients with HfrEF. The patients treated with S/V showed an improved LVEF, as well as improvements in most of the cardiac remodeling indices, like the LV end-diastolic volume, the LV end-systolic volume, the left atrial volume, and the LV mass index, as compared with patients treated with ACEIs or ARBs. Patients appeared to benefit more if treated with S/V as early as possible and for a duration of at least 3 months (15). A possible explanation for the reverse cardiac remodeling effect relies on the possibility that the neprilysin inhibitor fostered the reparative processes. In an experimental rodent model of progressive doxorubicin-induced cardiotoxicity, S/V offered greater protection against LV remodeling and dysfunction compared with valsartan (16).

Cardiac magnetic resonance (CMR) is the gold standard for ventricular dimension and ejection fraction assessment. All our patients had biventricular dysfunction in the acute phase, but, while LV impairment was clearly detected by echocardiography, RV dysfunction was detected only by CMR. However, EF reduction is just a small part of cardio-toxic damage (17). Early diagnosis of myocardial damage is crucial for its reversibility and the CMR seems to be the most effective tool to reach this aim because of its capability of tissue characterization (18). The T1 and T2 mapping CMR can easily highlight the additional signs of myocardial injury. In particular, native T1 values reflect the signals from the intracellular and extracellular compartments as well as intrinsic variances in tissue properties. An increased native T1 is useful for detecting the acute myocardial pathologies that can also occur in cardiotoxicities, such as edema, infarction, myocarditis, and subacute processes like diffuse fibrosis (19).

The reference for non-invasive recognition of focal fibrosis areas is LGE, but a limitation of this technique is the low sensitivity for diffuse fibrosis that is more frequent in patients with cardiotoxicity, especially in anthracycline cardiomyopathy (20). The T1 mapping can be considered as the early tissue markers of ventricular remodeling, whose increase was directly related to the administered dose and was inversely related to the exercise capacity, myocardial mass, and reduction in parietal thickness (21). All our patients had increased native T1 and ECV values, which are related to a diffuse increase in collagen content leading to a change in myocardial extracellular volume and resulting in diffuse fibrosis (22). The T2-weighted imaging can identify the presence of edema, which is secondary to acute myocardial inflammation and injury. Therefore, the increased native T1 and T2 values can detect an early myocardial inflammation, while elevated native T1 but normal T2 demonstrate subsequent interstitial fibrosis and remodeling (23). However, the utility of T2 maps in cardiotoxicity has not been thoroughly studied. Although they are very promising techniques, both have some limitations, mostly related to their dependence on physiological factors (for ex: age, sex, and heart rate) and on CMR protocols. Reference values should be individually validated in every radiological institution (24). In particular T2 maps are an ongoing matter of study in the CMR field and their utility in cardiotoxicity has not been thoroughly studied.

Implantable cardioverter defibrillators (ICDs) are indicated for the primary prevention of sudden cardiac death (SCD) in patients with reduced LV function (LVEF equal to or <35%). A subset of patients with cancer is at risk for SCD due to a variety of cardiac causes, including chemotherapy-induced cardiomyopathy. The data regarding the risk of arrhythmic death in these patients are very limited, but a study on the use of WCD in patients suffering from anthracycline cardiotoxicity showed a risk of malignant arrhythmia at around 7% in 3 months, which is significantly higher than in the general population with heart failure (9). An individual with cancer may have contraindications for permanent defibrillator implantation, including the potential reversibility of cardiomyopathy, an unclear prognosis for 1-year survival, and an increased risk of device infection related to some chemotherapies. Moreover, radiotherapy may interfere with the ICD function, and the presence of ICD can reduce the radiation dosing to the targeted tumor area. The WCD may protect the patients with cancer, who are at risk for SCD until an ICD can be safely implanted or until it has become unnecessary. One of the limitations of the WCD is the possible reduced compliance to continuously wear the device and the lack of pacing in the patients who are pacemaker-dependent (25). However, the median wearing time, reported in the most recent registry data, is higher than 23 h a day (26, 27). An interesting new feature of this technology is the recent integration of sensors that allow the physician to monitor the hemodynamic compensation and the patient's state of health as a whole. The WCD (LifeVest®, ZOLL, Pittsburgh, PA, USA) can obtain information about the average heart rate, the physical activity performed in daily steps, the body positions during days, the body angle, and the body position while the patient is reclined, indirect indices of physical capacity, and state of congestion. Our patients tolerated WCD with high compliance. While there were no spontaneous ventricular tachyarrhythmia events in this pilot evaluation, the use of the WCD in patients, who are actively receiving chemotherapy, is feasible and acceptable to these patients as demonstrated by the high compliance rates. In patients agreeing to undergo chemotherapy, non-invasively preventing the sudden cardiac death during periods of high short-term risk is appealing. Larger studies will be needed to clearly demonstrate the short-term and long-term benefits of such a strategy.

## Patient Perspective and Conclusions

Chemotherapy-induced acute heart failure could represent a potentially life-threatening side effect of many oncology drugs. Besides representing a clinical emergency, it could also negatively affect long-term patient outcome from an oncological point of view. In fact, a steadily impaired EF could limit the anticancer therapeutic options. Prompt and steady recovery of cardiac function has been a 2-fold relevance. All available therapeutic strategies should be implemented to get this goal. The presented case series highlighted the positive role of the early use of S/V in LVEF recovery in this clinical setting. The WCD should be considered in oncology patients when recovery of cardiac function is expected. A prospective evaluation of a larger size of the S/V effect in an oncology population is needed to confirm its retrospective and positive results.

## Data Availability Statement

The original contributions presented in the study are included in the article/supplementary material, further inquiries can be directed to the corresponding author.

## Ethics Statement

Written informed consent was obtained from all participants for their participation in this study.

## Author Contributions

MLC, KC, GS, JDM, AC, FS, and EV: study conception and case description. MLC, KC, GS, AC, GC, NM, CT, and IB: drafting of the manuscript or revising it critically for important intellectual content. All authors: final approval of the manuscript.

## Funding

This study received funding from Zoll (Pittsburgh, PA, USA) for the open access option only. The funder was not involved in the study design, collection, analysis, interpretation of data, the writing of this article or the decision to submit it for publication.

## Conflict of Interest

This study received funding from Zoll (Pittsburgh, PA, USA) for open access option only. The funder was not involved in the study design, collection, analysis, interpretation of data, the writing of this article or the decision to submit it for publication. The authors declare that the research was conducted in the absence of any commercial or financial relationships that could be construed as a potential conflict of interest.

## Publisher's Note

All claims expressed in this article are solely those of the authors and do not necessarily represent those of their affiliated organizations, or those of the publisher, the editors and the reviewers. Any product that may be evaluated in this article, or claim that may be made by its manufacturer, is not guaranteed or endorsed by the publisher.
